# Detection of a novel large fragment deletion in the alpha-globin gene cluster using the CNVplex technology

**DOI:** 10.3389/fgene.2025.1518392

**Published:** 2025-03-10

**Authors:** Jianfei Xu, Liang Hu, Lijuan Wen, Xianzhen Cao, Hongyan Xu, Qi Luo, Yuhong Long, Tingyu Ji, Lifang Sun, Fengxiang Wei

**Affiliations:** ^1^ Jiamusi University, Jiamusi, China; ^2^ Longgang District Maternity & Child Healthcare Hospital of Shenzhen City (Affiliated Shenzhen Women and Children’s Hospital (Longgang) of Shantou University Medical College), Shenzhen, China; ^3^ Laboratory of Shenzhen Children’s Hospital, Shenzhen, China

**Keywords:** thalassemia, α-globin gene, CNVplex, single-molecule real-time sequencing, deletion

## Abstract

**Objective:**

To describe the characterization of a novel deletion causing α-thalassemia.

**Methods:**

The proband was a 4-year-old boy who presented with abnormal hematological parameters identified during routine blood investigation conducted for a cold. Three common α-globin gene deletions, three mutations, and 17 mutations in the β-globin gene were detected using PCR-flow fluorescence hybridization. Next-generation sequencing (NGS) and CNVplex technologies were employed to identify potential rare pathogenic mutation types. The CNVplex technology leverages variations in the lengths of linkage sequences of differential sequences at the same locus to produce linkage products of varying lengths, thereby enabling the detection of multiple loci within the same system. The newly identified deletions were further validated using customized third-generation sequencing (TGS) and Sanger sequencing.

**Conclusion:**

In this study, hematological analysis indicated a potential diagnosis of thalassemia in the proband, characterized by typical microcytic hypodermic features. A novel 134-kb deletion in the α-globin gene cluster was identified in this proband using the CNVplex technology. This deletion encompasses the genes *HBZ*, *HBM*, *HBA2*, *HBA1*, and *HBQ1*. Furthermore, we confirmed the gene deletion through customized TGS testing and Sanger sequencing, allowing us to determine the size of the deletion. The results suggest that this represents a new deletion of 146 kb that has not been previously reported, and we hypothesize that this deletion is likely the primary cause of the α-thalassemia trait observed in the proband.

## Introduction

Among the types of thalassemia, α-thalassemia is primarily characterized by deletions and duplications of large genomic segments (Colosimo et al., 2011). The phenotype of α-thalassemia is directly related to the number of α-globin genes affected and can be classified into several categories: α^+^-thalassemia (-α/αα or αTα/αα), α^0^-thalassemia (--/αα or -α/-α), HbH disease (--/-α or --/α^T^α), and Hb Bart’s hydrops fetalis syndrome (--/--). Hb Bart’s fetal edema syndrome occurs in fetuses presenting with severe anemia, often resulting in fetal demise late in pregnancy or at birth (Piel and Weatherall, 2014). The routine test kits currently employed in the majority of hospitals are designed to target high-frequency variants within the local population, thereby failing to detect low-frequency variants. This limitation can pose challenges in clinical counseling and prenatal diagnosis (Li, 2017). With continuous advancement in genetic testing technology, the detection of novel mutations is expected to become increasingly easier and more efficient (Jiang et al., 2023). The CNVplex high-throughput linkage-dependent probe amplification (HLPA) technology is used to accurately detect deletion or duplication mutations in each exon of a target gene (or any deletion or duplication mutation in any non-duplicated target segment greater than 60 bp). The CNVplex technology is based on the use of a ligase-specific ligation reaction to make the target fragment through hybridization and ligation so as to introduce different lengths of non-specific sequences at the end of the ligation probe and to obtain different lengths of ligation products corresponding to different sites, and then through the use of universal primers with different markers of fluorescence through the PCR will be ligated to amplify the product, and then the PCR product on the sequencer to run capillary electrophoresis to separate out the different lengths of fragments. Subsequently, the PCR products were amplified using various labeled fluorescent primers. These products were then subjected to capillary electrophoresis on a sequencer to separate fragments of different lengths. The resulting capillary electrophoresis profiles were analyzed to identify the peaks corresponding to each site, allowing for the calculation of the copy number of the region of interest in determining the ratio of the target region peaks to the reference peaks (Liang et al., 2017; Wei et al., 2024). This test has been employed in the molecular diagnosis of other genetic disorders, such as congenital deafness and congenital heart disease (Huang et al., 2018; Hu et al., 2022). In this study, we identified a novel deletion of the α-globin gene using the CNVplex technology, determining the size of the deletion to be 146 kb through customized TGS for further validation and precise localization.

## Case presentation

The proband was a 4-year-old boy from Shenzhen, Guangdong Province, who presented to our hospital with symptoms of a cold. His hematological parameters were assessed using an automated hematology analyzer (XT-2000i, Japan), which indicated that he was suffering from microcytic hypodermic anemia. Specifically, he exhibited a mean corpuscular volume (MCV) of 60.8 fL (reference range: 76.0∼88.0 fL) and a mean hemoglobin concentration (MCH) of 20.1 pg (reference range: 24.0∼30.0 pg). Notably, the patient’s erythrocyte count was 6.17 × 10^12/L^ (reference range: 4.0∼5.50 × 10^12/L^). It is speculated that these findings may represent a compensatory response. Detailed hematological indices are presented in Table 1, and iron deficiency has been excluded. Therefore, we consider the proband to be at risk for thalassemia. Given the patient’s positive thalassemia phenotype, thalassemia screening was advised. For this screening, genomic DNA was extracted from peripheral blood using the DNeasy extraction kit (QIAGEN Inc., Valencia, CA, United States) and analyzed via PCR-flow fluorescence hybridization (Daan Gene, Guangzhou, China) including three α-thalassemia deletions--^SEA^ (NC_000016.9:g.215,400_234700del),-α^3.7^(NC_000016.9:g.223300_227103del), and -α^4.2^(NC_000016.9:g219817_(223755_224,074)del); three mutations (CD122(CAC>CAG) *HBA2*:c.427T>C, CD142 (TAA>CAA) *HBA*2:377T>C, CD125(CTG>CCG) *HBA*2:427T>C); and 17 mutations in the β-globin gene (CD41-42 (-CTTT) *HBB*:c.126_129del, IVS -2-654(C>T) *HBB*:c.316-197C>T, CD17 (A>T) *HBB*:c.52A>T, −28 (A>G) *HBB*:c.-78A>G, CD26 (G>A) *HBB*:c.79G>A, CD71-72 (+A) *HBB*:c.217dupA, CD43 (G>T) *HBB*:c.130G>T, −29 (A>C) *HBB*:c.-79A>C, Int (ATG>AGG) *HBB*:c.2T>G, CD14-15 (+G) *HBB*:c.45dupG, CD27-28 (+C) *HBB*:c.85dupC, −32(C>A) *HBB*:c.-82C>A, −30 (T>C) *HBB*:c.-80T>C, IVS-1-1 (G>T) *HBB*:c.92 + 1G>T, IVS-1-5 (G>A) *HBA*1:c.95 + 5G>A, CD31 (-C) *HBB*:c.94delC, and Cap+40–43 (-AAAC) *HBB*:c.-10_-7delAACA). The proband did not exhibit any abnormalities. Given his hematological parameters, there was a suspicion that he might be carrying a rare thalassemia gene. Consequently, after obtaining consent from the proband and his family, blood samples were collected for the targeted sequencing of the *HBA1*, *HBA2*, and *HBB* genes using NGS (MGI Tech Co., Shenzhen, China).

**TABLE 1:** Hematological data, serum ferritin, and iron levels of the family members.

Sample	Sex-age	RBC (×10^12^/L)	HGB (g/L)	MCV (fL)	MCH (pg)	*HBA* (%)	*HBA*_2_ (%)	HbF (%)	Serum ferritin (µg/L)	Serum iron (µmol/L)
Proband(II_1_)	M-4 years	6.17↑	124	60.8↓	20.1↓	97.4	2.6	0	40.67	16.6
Father(I_1_)	M-37 years	4.94	154	89.3	31.2	97.5	2.5	0	ND	ND
Mother (I_2_)	F-38 years	4.90	151↑	90.6	30.8	97.20	2.6	0.2	ND	ND
Sister(II_2_)	F-11 years	4.35	123	83.4	28.3	97.4	2.6	0	ND	ND

**FIGURE 1:**
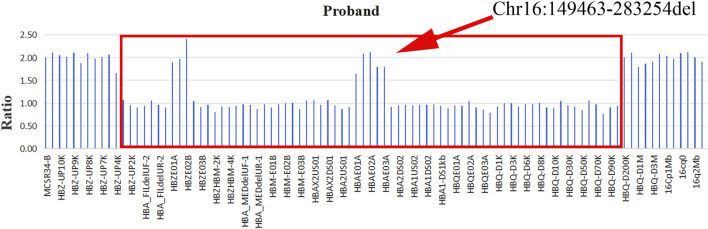
CNVplex analysis of the position of α-globin gene clusters deletion coordinates in proband.

No abnormalities were detected as a result of this testing. Based on the patient’s hematological parameters, it was presumed that she had a thalassemia trait (--/αα). Following informed consent from the proband and his family, blood samples were collected from the proband’s parents and sister and subjected to CNVplex testing (Suzhou Tianhao Biomedical Laboratory, Suzhou, China). The CNVplex technology was employed to detect deletion and duplication mutations in these samples, and point mutations were analyzed in conjunction with Sanger sequencing. The results revealed an unreported deletion of approximately 134 kb (Chr16:149463–283254) on the *HBA* gene of this proband (Figure 1). Limitations on the number of probes restricted the precise localization of the deletion, and testing of his family members did not yield any abnormal results. The proband was confirmed to have a new large segmental deletion.

**FIGURE 2:**
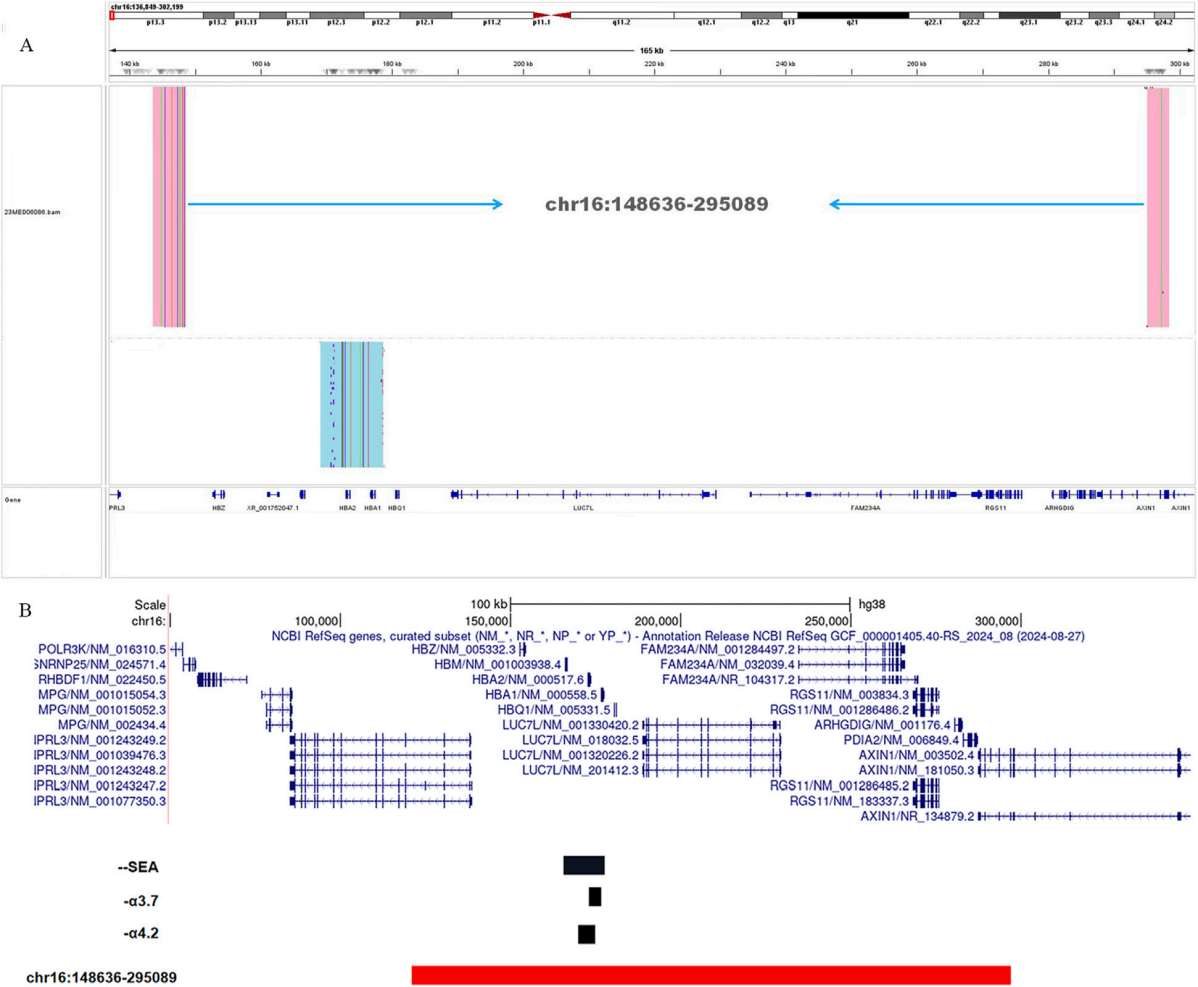
TGS analysis of the α-globin gene cluster of the proband **(A)**. Schematic representation of the telomeric region of chromosome 16 of the α-globin gene cluster. The novel deletion of the proband (Chr16: 148636-295089del) is indicated in red, and the black deletion indicates the three deletion types that are currently common in the clinic (--^SEA^, -α^3.7,^ and -α^4.2^). The novel deletion was compared with the three common deletion types --^SEA^, -α^3.7^, and -α^4.2^ **(B)**.

**FIGURE 3:**
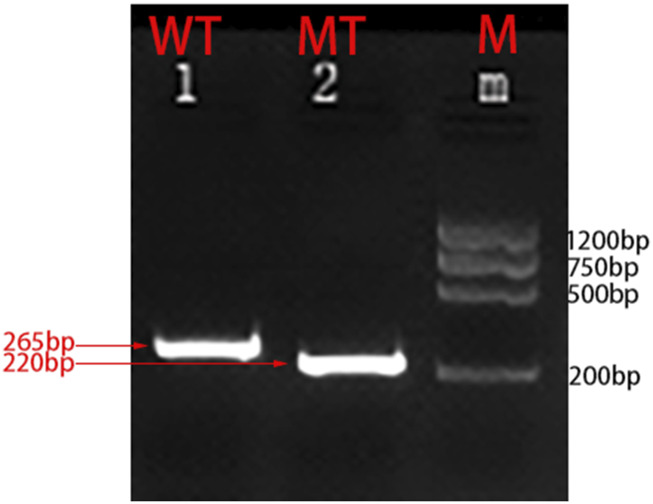
Molecular characterization of the large fragment deletion in this proband. Two primers specific for the α-globin gene cluster were designed. Amplification was successfully performed by GAP-PCR:F:GGGTGCTGTCCGCTTTCTA and R:GTGAGCCGAGATCCGAGGTC (mutant-type); F:GGGTGCTGTCCGCTTTCTA and R:AAGTAGAGTCCTGTTTCCAGGGTAG (wild-type).

**FIGURE 4:**
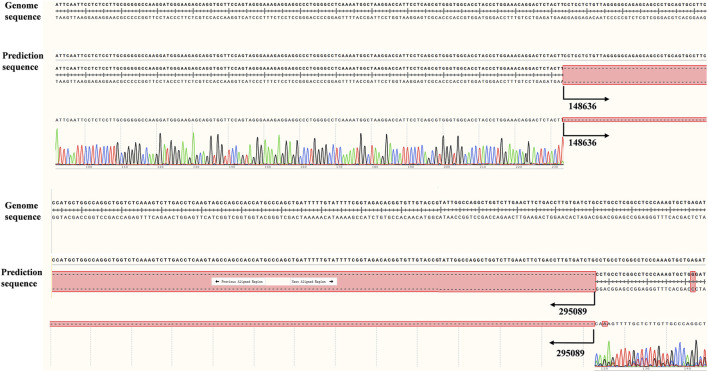
Difference between upstream and downstream breakpoints by TGS and Sanger sequencing.

To further validate the accuracy of the technique and clarify the exact location of the deletion, we employed long-read single-molecule real-time sequencing technology (SMRT) to analyze the proband. Preliminary results indicated that the *HBA* gene in this sample exhibited a haplotype of 1, which does not rule out the possibility of a deletion occurring outside the detection range. To accurately identify the precise breakpoints of the deletion in this sample, we customized and validated samples using long-read single-molecule real-time sequencing technology. Initially, primers were designed for the breakpoints based on the range of deletions detected by CNVplex. Amplification products were obtained through GAP-PCR targeting the specified region, and these products were subsequently subjected to TGS sequencing. The customized TGS validation revealed a deletion of approximately 146 kb in size within the α-globin gene of this proband (Figure 2A). Using a relatively accurate range estimation, primers were designed on both sides of the breakpoint, resulting in a unique GAP-PCR product (Figure 3). Sanger sequencing confirmed that the breakpoint was located at chr16:148636-295089 (GRCh38/hg38), indicating a deletion of 146,453 bp (Figure 4). Through database searches, including NCBI and HbVar, we concluded that the deletion represents an unreported large-band deletion of approximately 146 kb located on chromosome 16 (Chr16:148636-295089, GRCh38/hg38) (Figure 4). Subsequently, we obtained the registration number for the novel mutation in the IthaNet database (ID: 4110), which has been designated as a 146-kb deletion.

In comparison to the common types of α-globin gene deletions observed in clinical settings, the deletion fragment in this case is larger and includes genes such as *HBZ*, *HBM*, *HBA1*, *HBA2*, and *HBQ* (Figure 2B). Notably, both key genes associated with thalassemia, *HBA*1 and *HBA*2, are completely deleted, leading to the manifestation of α^0^-thalassemia. Additionally, based on the patient’s hematological characteristics, we noted a more pronounced decrease in MCV, MCH, and other markers when compared to the typical α-globin gene deletions, including -α^3.7^/αα, -α^4.2^/αα, and--^SEA^/αα (Xian et al., 2022).

## Discussion and conclusion

In the diagnosis of thalassemia, the presence of thalassemia-associated phenotypes is typically first assessed based on the patient’s blood count and hemoglobin electrophoresis. Subsequently, commonly employed thalassemia genetic testing techniques, such as Gap-PCR, PCR-RDB, and flow fluorescence hybridization, are used. However, the conventional assays currently employed in clinical laboratories are limited to detecting high-frequency variants that are specific to the local population. When there is a strong suspicion of a deletion that cannot be identified by conventional methods, we often resort to multiplex ligation-dependent probe amplification (MLPA) for detection. Nonetheless, the density of the MLPA probes restricts its ability to accurately pinpoint the deletion (Li et al., 2023).

After decades of development, next-generation sequencing (NGS) and single-molecule real-time (SMRT) sequencing have emerged as reliable tools for thalassemia, demonstrating both accuracy and stability. These technologies have been further validated in mass screening across various populations in China. NGS enables the detection of not only globin genes but also modifier genes such as *BCL11A*, *MYB*, and *KLF1*; however, it is generally limited by a read length of 300 base pairs (bp) (Wu et al., 2022; Li et al., 2023). While NGS offers high throughput, its sequencing results can be inaccurate in repetitive regions, guanine- and cytosine-rich regions, and highly homologous regions, primarily due to the short read lengths and the PCR amplification that occurs during the sequencing process. Furthermore, NGS struggles to differentiate between homologous genes such as *HBA1* and *HBA2*, as well as *HBB* and *HBD* (Zhou et al., 2023; Huang et al., 2024). In this study, NGS did not successfully detect the genotype of the sample. In contrast, the advantage of long-read single-molecule real-time sequencing technology over NGS lies in its ability to achieve read lengths of up to tens of kilobases. This technology allows for the direct sequencing of long-stranded DNA or RNA molecules without the need for fragmentation or amplification, thereby enabling the accurate identification of large deletions (Long et al., 2022).

Although TGS currently possesses significant technical advantages in diagnosing thalassemia, the high cost of equipment and the lengthy turnaround time for tests limit its widespread application in screening and diagnosing the condition (Liang et al., 2023). In this study, α deletion of approximately 134 kb on the α-globin gene was identified using the CNVplex technique; this deletion encompassed the *HBZ*, *HBM*, *HBA2*, and *HBQ1* genes. We hypothesize that the complete deletion of the *HBA1*/*HBA2* gene is the primary cause of the abnormal phenotype observed in this proband. Furthermore, we detected a deletion size of 146 kb using long-read SMRT sequencing technology. Notably, the 12-kb fragment that differed from the CNVplex results contained the AXIN1 gene, which is a component of the AXIN1–HIPK2–TP53 complex that regulates cell growth, apoptosis, and development (Li et al., 2007).

The CNVplex technology can be used to detect deletion or repeat mutations in each exon of a target gene. However, this technology is not sufficiently accurate to estimate the extent of large deletions in the α-globin gene when compared to SMRT, primarily due to the limitations associated with the number of probes available. In this study, the SMRT sequencing technology was employed based on the approximate deletion extent provided by CNVplex for further detection. Without this approximate range of deletions for the subject, the SMRT sequencing technology identified only one *HBA* haplotype in the sample. We speculate that the types of mutations detectable by the SMRT sequencing system are constrained by the system’s limitations in analyzing amplified LD-PCR products, which typically range from 10 to 20 kb. Consequently, if the triplex system fails to amplify this disease-causing haplotype, larger fragment deletions exceeding 80 kb may remain undetected.

Based on this study, we conclude that the CNVplex technique offers significant advantages, including its relative operational simplicity and its enhanced capability to detect various large-band deletions present in the α-globin gene associated with thalassemia. Thus, we assert that CNVplex serves as an effective primary screening method for identifying rare and novel α-thalassemia deletions. However, to further elucidate the true coverage of the α-globin gene cluster, technologies such as long-read SMRT sequencing are necessary. Our findings from the hematological tests, in conjunction with the SMRT sequencing results, suggest that this deletion represents a *de novo* deletion in the α-globin gene, approximately 146 kb in length, and that its clinical presentation may be more severe compared to the--^SEA^/αα genotype.

## Data Availability

The datasets presented in this study can be found in online repositories. The names of the repository/repositories and accession number(s) can be found at: https://www.ithanet.eu/db/ithagenes?ithaID=4110, 4110.
