# Supplementary figures and images for: Detection of a novel large fragment deletion in the alpha-globin gene cluster using the CNVplex technology
Source: Front Genet. 2025 Mar 10;16:1518392. doi: 10.3389/fgene.2025.1518392 (PMC11931057; doi:10.3389/fgene.2025.1518392)

Supplemental figure. Validation of STR locus affinities in probands and their families.

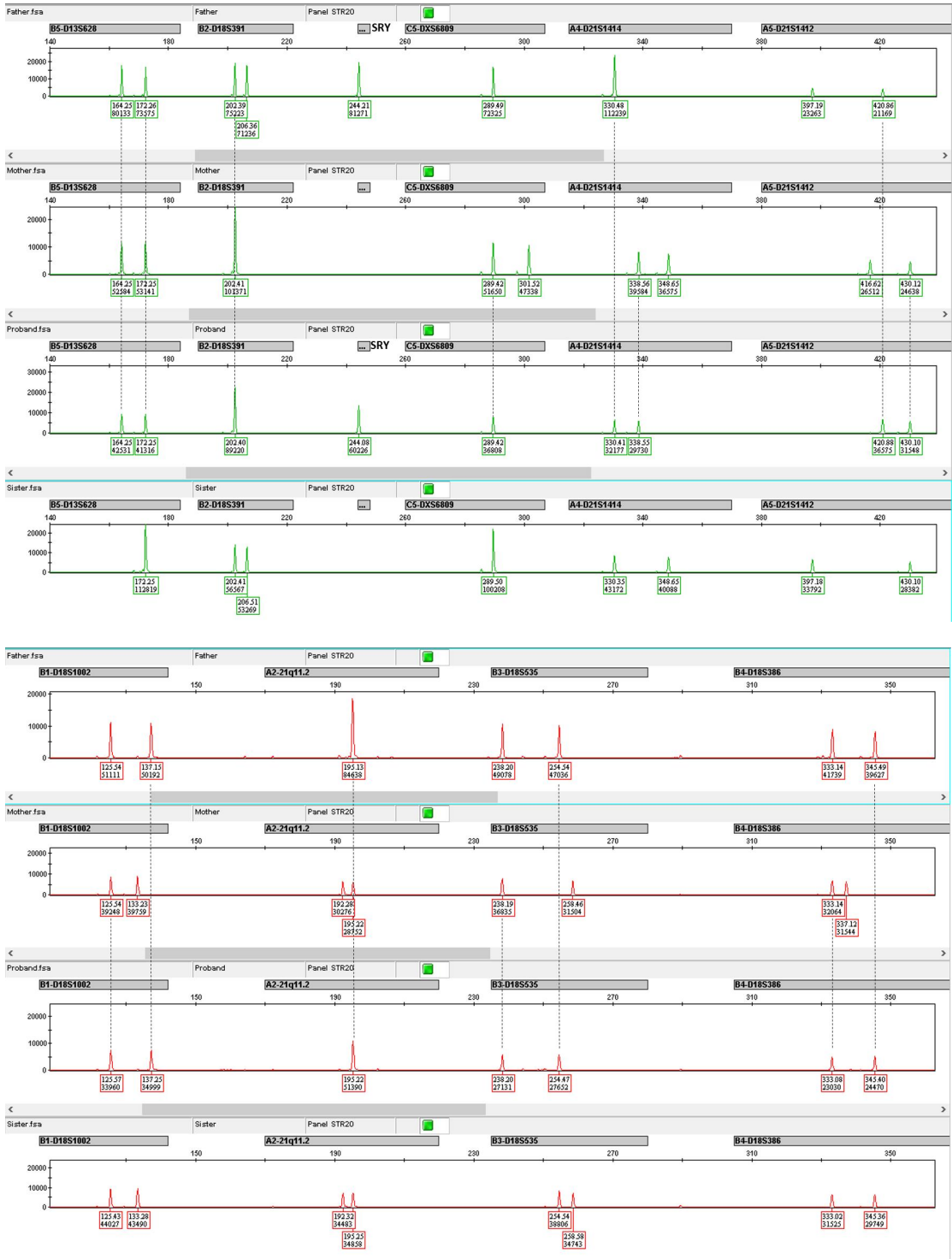

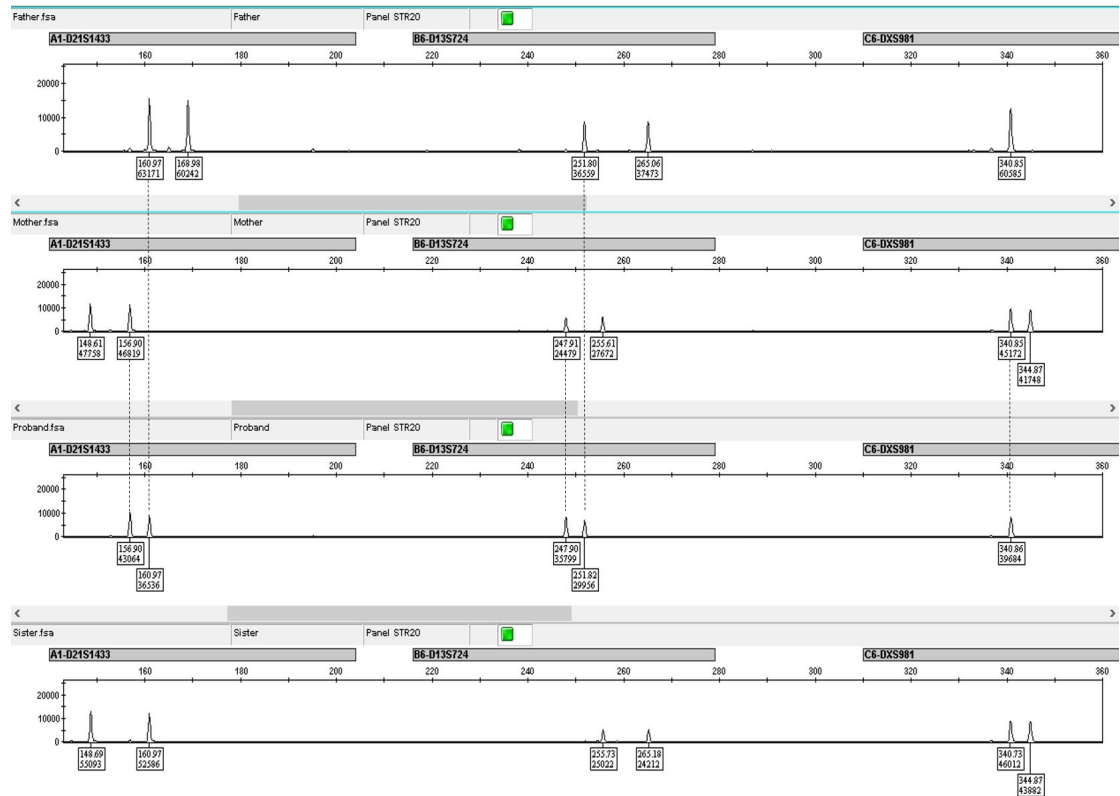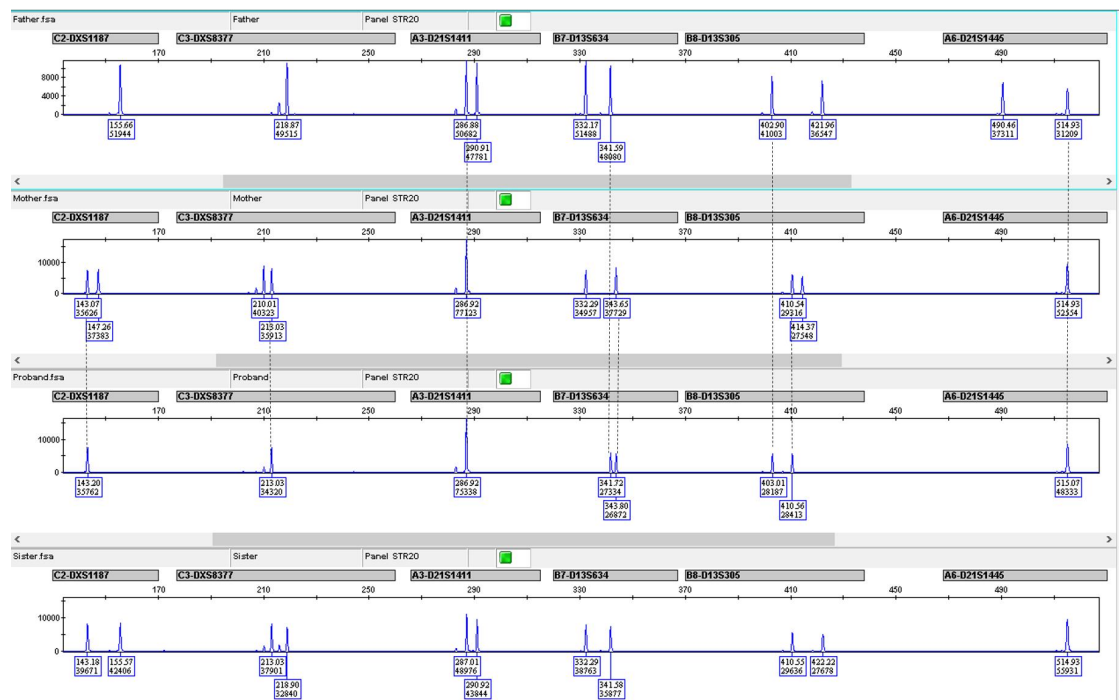

Supplement: Supplementary file 1 [file DataSheet1.pdf]
